# The Suppression of Immune System Disorders by Passive Attrition

**DOI:** 10.1371/journal.pone.0009648

**Published:** 2010-03-16

**Authors:** Sean P. Stromberg, Jean M. Carlson

**Affiliations:** 1 Biology Department, Emory University, Atlanta, Georgia, United States of America; 2 Physics Department, University of California Santa Barbara, Santa Barbara, California, United States of America; Albert Einstein College of Medicine, United States of America

## Abstract

Exposure to infectious diseases has an unexpected benefit of inhibiting autoimmune diseases and allergies. This is one of many fundamental fitness tradeoffs associated with immune system architecture. The immune system attacks pathogens, but also may (inappropriately) attack the host. Exposure to pathogens can suppress the deleterious response, at the price of illness and the decay of immunity to previous diseases. This “hygiene hypothesis” has been associated with several possible underlying biological mechanisms. This study focuses on physiological constraints that lead to competition for survival between immune system cell types. Competition maintains a relatively constant total number of cells within each niche. The constraint implies that adding cells conferring new immunity requires loss (passive attrition) of some cells conferring previous immunities. We consider passive attrition as a mechanism to prevent the initial proliferation of autoreactive cells, thus preventing autoimmune disease. We see that this protection is a general property of homeostatic regulation and we look specifically at both the IL-15 and IL-7 regulated niches to make quantitative predictions using a mathematical model. This mathematical model yields insight into the dynamics of the “Hygiene Hypothesis,” and makes quantitative predictions for experiments testing the ability of passive attrition to suppress immune system disorders. The model also makes a prediction of an anti-correlation between prevalence of immune system disorders and passive attrition rates.

## Introduction

The immune system provides protection from diseases ranging from intestinal parasites to viruses and even cancers. The immune system is also the cause of many other types of disease, like autoimmune diseases and allergies. There is a large body of evidence [1]–[3], ranging from epidemiological [4], [5] to animal model experiments [6], [7], showing that exposure to the diseases that the immune system fights provides protection from the diseases that the immune system causes. The paradoxical protection conferred by pathogenic infections against immune system disorders is often referred to as the “Hygiene Hypothesis” [1], [8]. Understanding the mechanisms of this protection has important clinical consequences.

There are several proposed mechanisms through which pathogenic infections may provide protection from immune system disorders. The mechanisms receiving the most attention are competition for antigen [9] and bystander suppression [10]. Competition for survival factors (the topic of this paper) has also been proposed [11]. Quantitative models are essential in assessing the strength and importance of the various candidate mechanisms of protection. Infectious diseases have also been shown to directly trigger certain autoimmune diseases [11]. While there are many examples of this effect [12], this is not a general feature of infectious diseases. Most people for example get sick with an infectious disease one or two times a year, yet even with this frequency of infection, autoimmune disease remains comparatively rare.

In this paper we quantify a specific mechanism by which infectious diseases may suppress immune system disorders. This mechanism is the increased competition for homeostatic survival factors generated by the addition of new cells to the homeostatic niche upon infectious disease exposure. In this paper, niche refers to the set of cells competing for the same growth factor. This increased competition following infection is also referred to as passive attrition [13]–[15]. Passive attrition contributes to long term decay of immunological memory. As new cells are added to various niches of the immune system all existing sub-populations will decrease in number, making room for the new cells.

The mechanism of passive attrition can not only lead to loss of specific memory over time, but also act in a beneficial manner by suppressing immune system disorders such as allergies and autoimmune diseases. The model that we present in this paper generates experimental predictions on both an epidemiological level and that of individual animal experiments. It also offers a reinterpretation of past observations.

### Model

**Figure 1:**
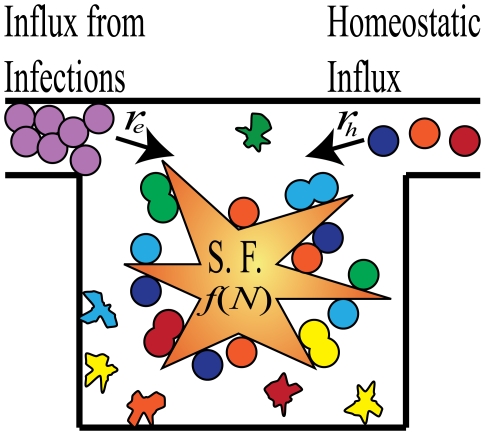
Illustration of the dynamics of homeostatic regulation. Cells enter the system from either infections 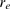 or through homeostatic influx 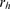, which is zero for some niches. Survival factors (S.F.) regulate the total number of cells in the niche by either inhibiting cell death or inducing cell division. The rate of stimulation by survival factor for each cell, 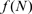, is a function of the total number of cells in the niche, 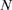.

The maintenance of a population of cells capable of either dividing or dying requires homeostatic regulatory mechanisms. The population could be maintained by an influx of new cells or by mechanisms that control the death or division rates of the populations. The regulatory mechanisms prevent both unconstrained growth (cancer) and decay of an essential cell type. The homeostatic regulation typically comes in the form of competition for survival factors, Figure 1. Competition provides a stabilizing mechanism for population size: too many cells and some will not have enough access to the survival factors, too few cells and there will be an abundance of survival factors allowing proliferation of the existing population.

**Figure 2:**
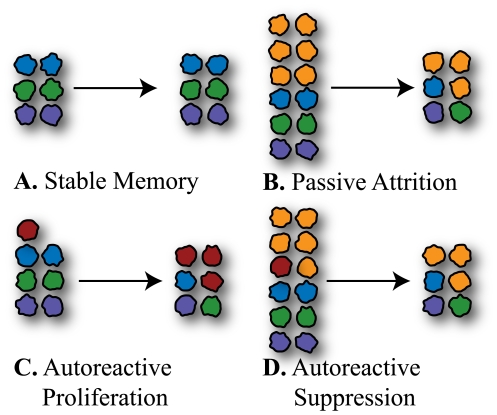
Illustration of the effects of passive attrition. **A.** Without an influx of new cells sub-populations are stable in number. **B.** With an influx of new cells the competition for survival factors is increased and all populations are reduced in number. This is referred to as passive attrition. **C.** Autoreactive cells (red) can be stimulated to divide by self-antigens. This gives them a competitive advantage over the other sub-populations in the niche. **D.** If the influx of new cells is large or the antigenic stimulation rate is small, the autoreactive population can experience passive attrition. In a filthy environment the influx of new cells from infections will be large, suppressing the growth of autoreactive populations. In the more sterile environment represented in **C.** this suppressive effect is absent.

The survival factors could be chemical signals such as interleukins or growth factors, or a constrained physical volume necessary to maintain the cell type. The group of cells that compete for the same set of factors is referred to as the niche. The niche may be shared by many sub-populations of cells such as antigen specific memory cells from previous infections. Studying the homeostatic mechanisms tells us the long term fate of these sub-populations. Though the total number of cells in the niche may remain constant over time the sub-populations could decay, remain constant or even grow. Figure 2A depicts a stable sub-population of cells. Figure 2B shows a sub-population of cells in a niche that is being added to by a source of new cells. In this case the pre-existing sub-populations of cells decay. This type of decay is called passive attrition.

In this paper we are interested in sub-populations of cells that can both be stimulated to divide by survival factors and by self-antigen. Left alone these cells would outcompete the other cells of the niche through their increased division rate from the self antigen, Figure 2C.

Figure 2C represents a scenario in a sterile environment where there is no passive attrition to suppress the initial growth of the autoreactive cell. In Figure 2D we consider a filthy environment where there is a large influx of new cells from immune responses. Here the autoreactive cells would also suffer passive attrition and could be suppressed. The possibility of suppression depends on how autoreactive the cells are, and on the rate of influx of new cells to the system.

**Figure 3:**
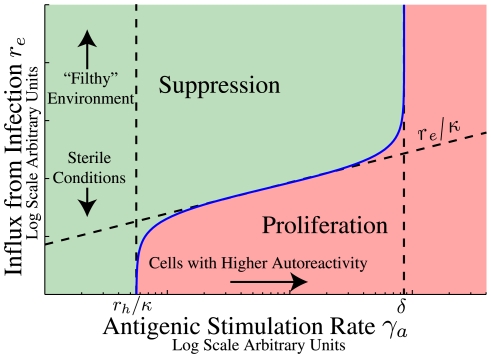
Illustration of a threshold for suppression by passive attrition. This threshold is defined by Eq. 6 separating conditions for suppression (green region) and proliferation (pink region) of autoreactive cells. The vertical axis is the influx from infection 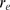. This quantity is typically controlled by the external environment and is expected to be proportional to the infection rate. The lower portion of the figure represents cells in a more sterile environment and the upper portion of the figure a filthy one with frequent infections. The horizontal axis is the antigenic stimulation rate 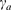 for a small population of autoreactive cells 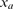. Cells with antigenic stimulation rate less than 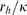 (the homeostatic influx divided by the number of cells in the niche at equilibrium) are always suppressed though for some niches 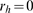. No populations with 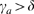 (where 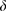 is the apoptotic rate under high levels of competition for survival factor) can be suppressed by passive attrition because the division rates of these cells (from autoantigen exposure) are large enough to maintain the population even in the absence of survival factors.

Figure 3 shows a characteristic result of the model presented in the Methods section. Here we show when an autoreactive population will be suppressed considering two variables: the influx of new cells (vertical axis) and the rate of antigenic stimulation of the autoreactive population (horizontal axis). The bottom of the graph represents sterile conditions and the top, filthy. The left portion of the graph is low autoreactivity of a clone of cells and the right is high autoreactivity. The bottom right is therefore highly autoreactive cells in a sterile environment that are bound to proliferate while the top left of the diagram is cells with low autoreactivity suppressed by a filthy environment.

Upon measuring the stimulation rate for an autoreactive population this type of diagram shows the conditions that will either suppress that population or allow it to proliferate. Measuring the self-antigenic stimulation rate and tracing up to the blue curve gives the critical rate of influx from infection. If the rate of influx of new cells exceeds this critical rate the autoreactive population will decay. The rate of influx of new cells could be controlled experimentally by either active transfer of cells or by infections.

The specific shape of the curve in Figure 3 depends on the details of homeostatic regulation. In the Methods section we derive quantitative mechanistic models of homeostasis of CD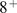 memory T cells, and for cells competing for interleukin 7. In Results we present the quantitative predictions of the model for these cell types and for the physiology of both humans and mice.

## Methods

We first present a general mathematical model of homeostasis without explicit definition of the regulatory mechanisms. This technique was previously used by Antia et al. [13], to show that passive attrition is a general property of homeostasis. We extend this result to show that suppression of autoimmune disease by frequent infection is also a general property of homeostasis. In the subsection CD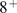 Memory T Cells, we explicitly model the regulatory mechanisms of the niche of T cells competing for interleukin 15 (IL-15), the niche that contains CD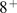 memory T cells. In the subsection following that titled The IL-7 Niche, we model the niche of cells competing for interleukin 7 (IL-7). This niche contains naive T cells and CD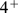 memory T cells. These models are calibrated for both humans and mice to provide quantitative predictions. Throughout we are modeling the average expected behavior, assuming well mixed populations in the niches.

Our general framework for homeostatic regulation does not consider systems that have multiple stable values for total cell number. An example of such a system would be long-lived, non-dividing cells, with number below the maximum population size of the niche. Systems such as this are not homeostatically regulated, and adding more cells to the niche has no effect on the cells already occupying it.

In general, a differential equation for the population dynamics of cells under homeostatic regulation has the form:
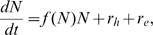
(1)where 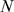 is the total number of cells in the niche, all competing for the same survival factors. The dynamics of this equation are pictorially represented in Figure 1. The different colors of cells in Figure 1 represent different antigen specificities.

The homeostatic influx 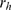 and the influx from infection 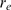 represent influxes of new cells, from homeostatic sources and antigenic stimulation, respectively. In the absence of any antigenic stimulation it is assumed that 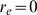. The influx from antigenic stimulation typically equals the product of the infection rate and the number of new memory cells per infection. The homeostatic influx 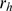 represents new cells which arise from homeostatic sources such as thymic output. For CD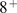 memory T cells, the niche is not likely shared with naive T cells and 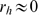, while for CD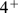 memory T cells, there is competition with naive T cells, and 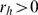 [16].

The rate 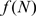 in Eq. 1 gives the homeostatic regulation of death and division. 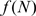 is called the attrition rate for reasons discussed below. This rate must be a function of the total number of cells in the niche in order for the homeostatic equilibrium to be stable. The more cells in the system the greater the level of competition for survival, and the lower the value of 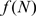. This gives us the requirement:
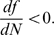
(2)The homeostatic equilibrium 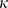 is the limiting number of cells that the system reaches when there is no antigenic stimulation, i.e. when 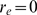. The homeostatic equilibrium is defined mathematically as:
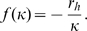
(3)Substituting Eq. 3 into Eq. 1, along with 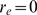 yields the stable (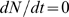) solution 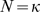. When 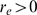 the cells generated by antigenic stimulation bring the total number above the homeostatic equilibrium, 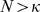. This reduces the homeostatic renewal, such that 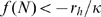. Except in lymphopenic conditions (where homeostatic proliferation can occur to refill the system) the attrition rate satisfies 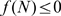.

The dynamics in Eq. 1 are represented pictorially in Figure 1. Cells enter the niche either from infections (rate 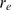) or from homeostatic sources (rate 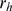) and compete with each other and the cells already occupying the niche for the limited amount of survival factors. The survival factors could either act by initiating cell division or by inhibiting cell death.

After the completion of an immune response there will be a sub-population of antigen specific memory cells added to the niche 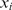, where 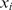 is the number of cells specific to antigen 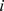. The different sub-populations are unique in their antigen specificity but not in their ability to compete for survival factors. The negative value of 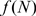 under the addition of new cells has consequences for the dynamics of these sub-populations. Since these populations share the same niche they will have the same homeostatic regulation term. However, these cells are not restimulated antigenically or added to appreciably from homeostasis, so the equation describing the time evolution of an individual sub-population lacks a source term:
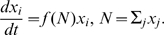
(4)(Repeated exposures would require an additional term for antigenic stimulation which we do not consider here as it would complicate the analysis but not alter the results.) If there is no influx of new cells (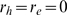) then 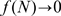 at equilibrium, and the individual memory cell populations are sustained indefinitely (ignoring stochastic effects, the subject of future research). With either 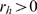 or 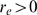, 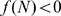 and the subpopulation will experience “passive attrition” [13]–[15], an exponential decrease in cell number over time with the rate 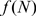, hence the term “attrition rate” for 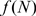.

**Figure 4:**
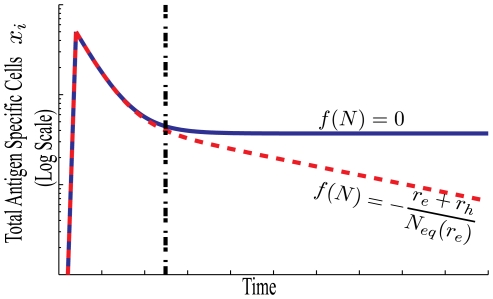
Memory formation with and without passive attrition. For both scenarios the number of antigen specific cells quickly rises during an immune response, then rapidly decreases until the total cell number is approximately at equilibrium, 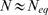, as indicated by the black dashed line. The blue curve illustrates the case with no passive attrition, where 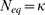, and 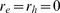. CD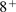 memory in a sterile environment is representative of this (blue) scenario. The red curve illustrates the scenario where new cells are frequently added to the niche shared by the specific memory, causing the number of antigen specific cells to decline over time.

Typical memory scenarios are shown in Figure 4. Antigen specific cell number 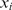 grows rapidly over the course of a few days in response to an infection (not modeled here). After the infection is cleared there is rapid cell death and memory formation until the total number of cells 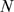 returns to a value near 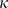 (at the time indicated by the dashed black line). The cell populations will then experience attrition with rate 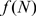.

The decrease of specific memory over time is a result of infections or influx of new cells raising the total number of cells and hence the level of competition for survival factors. CD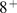 memory T cells in a sterile environment can survive indefinitely, as 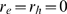, and therefore the rate of attrition 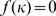 [16]. However, for the IL-7 niche, the influx of new naive T cells to the niche should contribute to the passive attrition of CD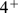 memory T cells and may be responsible for the observed bi-phasic decay [16].

We refer to sub-populations of cells that respond to either native antigen or allergen as simply autoreactive. In this case there will be an additional term (first term on the right hand side) for antigenic stimulation:
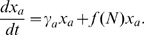
(5)The antigenic stimulation rate 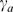 is the rate of cell division from stimulation by self-antigen. It is typically a complicated function involving competition for antigen, tolerance mechanisms such as regulatory T cells, and physiological changes in antigen presentation from inflammation and tissue damage.

We are interested in the behavior of a very small number of cells, before disease, and specifically whether the cell population proliferates or is suppressed. The antigenic stimulation rate 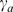, is the limiting value of this more complex rate, in the low cell number limit. The antigenic stimulation rates of different clones of cells will differ. The subscript on 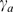 denotes the different growth rates for the different clones, 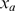.

There are two opposing rates for autoreactive cells, the rate of attrition 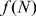 which acts to reduce their number, and antigenic stimulation 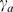 which causes proliferation and eventually disease. Depending on which of these rates is larger there are two possible outcomes for populations of autoreactive cells:
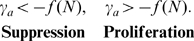
(6)Suppression results in exponential decay of any new population of autoreactive cells. Proliferation results initially in an exponential growth and could eventually lead to disease.

**Figure 5:**
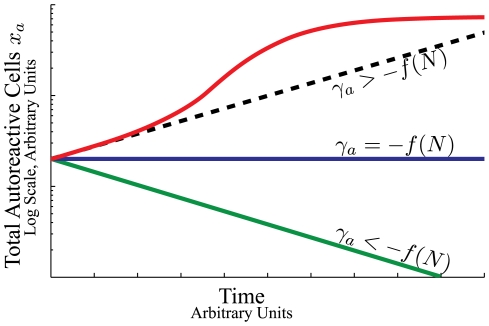
A schematic illustration of a small population of autoreactive cells 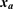. These cells can either be suppressed if 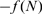 is large enough (green line) or experience exponential growth (black dashed line). If the cell population becomes large other factors will alter the growth rate such as feedbacks from inflammation and tolerance mechanisms, illustrated schematically in red.

In the absence of influx from infection (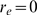), autoreactive cells with 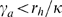 will be suppressed. Influx from infection decreases 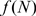, increasing the range of 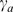 values that result in suppression. Figure 5 shows the possible scenarios for growth or decay of a small population of autoreactive cells.

The likely scenario consists of first an autoreactive cell escaping negative selection by not experiencing all self and environmental antigens as an immature cell. As this cell matures it enters the naive population where it may be stimulated by self-antigen or allergen. The antigenic stimulation causes the cell to proliferate into a small number of autoreactive memory cells. These autoreactive memory cells may still require survival factors to persist or proliferate. If this is the case, increasing the level of competition for survival factors can suppress this cell clone and thereby prevent development of disease.

The suppression of autoreactive cells in this manner is accompanied by the passive attrition of memory populations. Larger values of the attrition rate 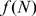 both suppress populations with greater ranges of antigenic stimulation rates 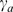, and causes more rapid loss of immunological memory. Conversely, for long-term stable memory populations there must be a low value of the attrition rate 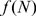, and thus populations with a greater ranger of 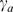 values will proliferate.

The inequality in Eq. 6 defines a boundary between suppression and proliferation that is a function of the rate of infection. Figure 3 illustrates a pedagogical example. The value of 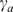 is a property of the cell and 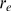 is typically a property of the external environment. For a clone of autoreactive cells 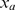 described by an antigenic stimulation rate 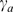, the boundary defines the minimum level of influx from infection needed to suppress that clone. For clones with large values of 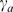 it is possible that there is no value of 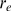 large enough to suppress them. Similarly it is possible that all 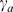 values below a certain limit might be suppressed by homeostatic sources of attrition, though this is not a common feature of all niches. Conversely, if we are considering an external environment that is well characterized by a particular value of 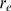, the boundary in the figure defines the lower limit of autoreactivities we are likely to find in that external environment. A low 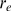 value corresponds to a more sterile environment while a large 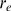 value is associated with a “filthy” environment. In later sub-sections we fit curves to data for human and mouse CD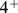 and CD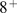 memory T cells to make quantitative predictions for these boundaries.

### The Low Infection Rate Limit

We can find the asymptotic behavior of passive attrition and autoreactive suppression in the limit of infrequent infections, i.e. low 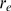. The equilibrium total number of memory cells for a given rate of infections is given by 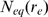. This is simply the value of 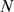 for which the right hand side of Eq. 1 is equal to zero:
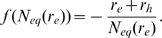
(7)If the infection rate is zero then 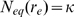, the homeostatic equilibrium. If we consider the case where the infection rate is small enough that the correction to 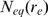 is insignificant compared to 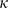 we have 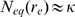. In this limit, Eq. 7 reduces to the form derived by Antia et. al [13]:
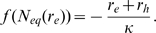
(8)This gives an exponential rate of decay of existing memory populations that is proportional to the sum of the rates of new cell incorporation:
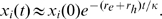
(9)We also have the condition for suppression in the limit of low influx from infection 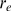:
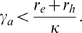
(10)As can be seen from this equation, clones with 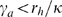 are always suppressed (since 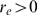).

For CD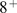 memory T cells the homeostatic influx 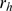 equals zero and there is no lower limit on ability of autoreactive cells to proliferate in sterile conditions. The asymptotic behavior of the boundary separating the regions of suppression and proliferation therefore follows approximately the curve 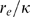 then converges to the vertical line of 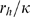. This asymptotic behavior can be seen in Figure 3 for the lower antigenic stimulation rate 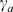.

The asymptotic result shows that addition of new cells to a homeostatic niche is a mechanism for suppressing or eliminating autoreactive cells with low antigenic stimulation rate 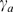, and that it is a common feature of homeostatic regulation. For larger values of 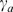 it may not be possible to satisfy Eq. 6. This is shown and discussed in the following sub-sections where we model the homeostasis of cells in the IL-15 regulated niche (CD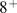 memory T cells), and cells in the IL-7 niche (CD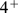 and naive T cells) respectively. There we also give quantitative predictions for the range of antigenic stimulation rates 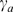 that will be suppressed in environments characterized by the rate of influx from infection 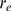.

### CD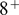 Memory T Cells

The best understood homeostatic regulation scheme in the mouse and human immune systems are the CD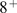 memory T cell pools [17]. These cells are differentiated from effector memory by the presence of high levels of CD122 on the cell surface. The CD122 protein is part of a receptor for IL-15. In the absence of IL-15 the CD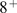 memory T cells can not survive. Other cell types are typically unaffected in the IL-15 knockout mouse [18] showing that the niche is not shared and that competition between the cells of this niche for IL-15 should have little effect on other cell types. Additionally we know that in a sterile environment memory populations in this niche are stable yielding 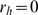 [19].

At homeostatic equilibrium the total number of cells remains constant. Since there is no homeostatic influx of new cells to this pool (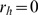), both the homeostatic division rate 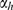 and the homeostatic death rate 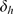 are therefore equal. With CSFE staining and other techniques it has been observed for mice that the homeostatic division rate is approximately once every 2–3 weeks, meaning 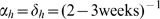 [20].

To discern whether IL-15 inhibits apoptosis or stimulates division, we consider the two possible cases separately. Inhibition of apoptosis is described by:
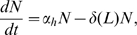
(11)where 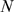 is the population size, the first term on the right hand side represents increases in the population due to division, and the second term represents decreases due to apoptosis. The quantity 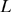 is the concentration of IL-15 and the apoptotic rate 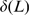 decreases with increasing 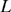. Judge et al. [18] placed CD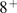 memory T cells in an IL-15 saturated solution. In the saturated environment we would expect 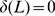, and if Eq. 11 were the correct description we would see the proliferation rate of the population equal to 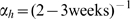. Instead the population was observed to double in less than three days which rules out Eq. 11 as a valid model. From this we conclude that IL-15 does not simply inhibit apoptosis.

Next we consider the stimulation of division by IL-15, described by the equation:
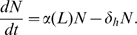
(12)Now the division rate 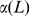 is a function of IL-15 concentration 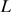, and increases with increasing 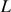. Another experiment by Judge et al. [18] transplanted CD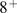 memory T cells into IL-15 knockout mice. In the absence of IL-15 and stimulating antigen, 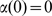, Eq 12 predicts a decay in cell number with rate 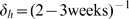. The observed decay took place over approximately 2 weeks [18] in agreement with the model. This implies that to a first approximation IL-15 stimulates division.

**Figure 6:**
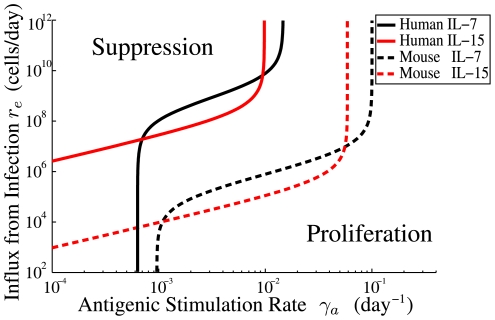
Boundaries discriminating between autoreactive cell populations that are suppressed vs those which proliferate, for humans (solid lines) and mice (dashed lines). The condition for suppression is given by Eq. 19 and 25 and the numerical values of the parameters are found in Table 1. The features of these curves are discussed in the caption to Fig. 3. CD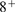 memory T cells belong to the IL-15 niche (red lines). The model predicts that for CD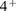 T cells which are in the IL-7 niche (black lines), all autoreactive populations 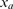 with antigenic stimulation rates 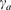 below 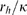 are suppressed by the homeostatic influx of naive T cells. Passive attrition can not suppress autoreactive cells with 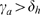.

We can compare the homeostasis expressed in Eq. 12 with our general model of suppression to obtain asymptotic behavior of the boundary separating suppression from proliferation, described by Eq. 6. Our previous requirement that 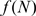 be a decreasing function of 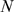, requires that 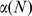 also be everywhere decreasing. Physically, this corresponds to the concentration of 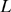 being lower the more cells there are competing for it. This gives us (from Eq. 6) the conditions for suppression:
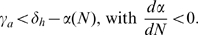
(13)There can therefore be no suppression by passive attrition for cells with 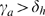. Physically, this corresponds to cells that can sustain their number through antigenic stimulation alone (characterized by large 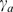) and do not require homeostatic signals for survival. The condition for suppression of autoreactive cells, as 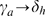, requires that 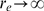. This asymptote is drawn explicitly in Figure 3 and is evident in the plots of Fig. 6.

The asymptotic behavior for the condition of suppression of autoimmune disease by passive attrition is given by only two parameters, 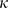 the homeostatic equilibrium number of cells in the niche, and 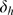 the homeostatic death rate of the population. Connecting the low 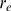 behavior, Eq. 10, with the behavior as 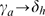 requires a more detailed model of the competition for IL-15.

Biologically, IL-15 is typically presented to CD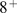 memory T cells by dendritic cells. The IL-15R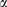 receptor on dendritic cells binds to IL-15 and presents it to the CD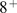 memory T cells where it binds to the CD122 molecule initiating signaling.

A rate equation that captures the correct asymptotic behavior in both limits and has an interpretation relating to competition for growth factor is a saturating function:
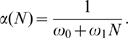
(14)This rate equation has the physical interpretation that the inverse of the rate, 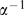, is the expected waiting time for stimulated division, and the waiting time is a linear function of 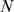. The shortest possible physiological waiting time is given by 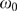, and in a system with more cells, the waiting time increases linearly as the competition for growth factor among cells increases with proportionality constant equal to 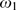. (The denominator in Eq. 14 can be viewed as the first order approximation of a more complicated function of waiting time 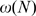.)

Inserting this into the population dynamics equation (Eq. 1 with 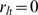) yields the homeostatic equation:
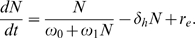
(15)This equation is functionally equivalent to the equation used by Utzny and Burroughs [21]. The homeostatic equilibrium (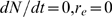) value 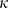 is given by:
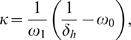
(16)and the equilibrium total number of memory cells in the presence of infections, 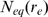 is given by:
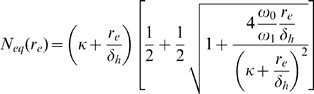
(17)

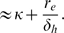
(18)The approximate form can be seen by working out the square of the denominator 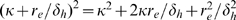. If any of these three terms is far greater than the numerator, the approximate form is valid. Substituting Eq. 16 for 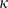 in the second term shows us that the approximate form is valid when the expected lifetime of a cell is much larger than the shortest time to division: 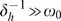. The approximation is not sensitive to the values of 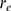 or 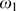. Experimentally 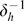 is on the order of weeks while 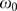 is approximately a day. The approximation is therefore a good one.

This gives us a functional form for the condition for suppression of autoreactive populations:
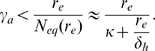
(19)This relation for autoreactive suppression by passive attrition relies on just two parameters: the homeostatic equilibrium 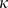, and the homeostatic death rate 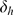. The right hand side of Eq. 19 is equal to the attrition rate 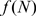 of the various memory populations in the niche, not being re-stimulated. This provides an observable quantity to test the model.

Equation 19 is plotted for both mice and humans in Results with physiological values for the parameters.

### The IL-7 Niche

The niche for CD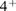 T cells is more complex than that for CD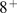 memory T cells. The Th1 and Th2 CD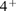 memory T cells share their niche with naive CD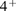 and naive CD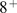 T cells. While cells in this niche may require both IL-7 and MHC contact, competition for MHC contact is not likely a limiting factor in homeostatic survival due to its ubiquity.

Assuming that the action of IL-7 is similar to that of IL-15 we have an equation similar to Eq. 15, but with an additional term for homeostatic influx of T cells entering the niche from the thymus, 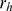:
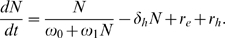
(20)The values of 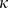, 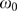, 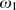, and 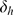 are different for the cells belonging to the niche competing for IL-7, than they were for CD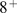 memory T cells. In this niche the total number of cells at homeostatic equilibrium 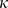, is the sum of the number of CD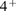 memory, CD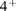 naive, and CD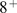 naive T cells at homeostatic equilibrium, since they are all competing for IL-7.

The rate of homeostatic homeostatic influx 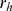 leads to passive attrition of CD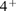 memory cell populations, even in sterile conditions. For mice the rate of attrition of CD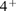 memory populations in a controlled environment has been measured to be around 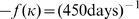 [16]. We know from Eq. 1 and 4, that the rate of attrition in the absence of influx from infection 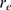 is given by 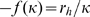, so:
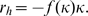
(21)The numerical values for mice and humans are presented in Table 1. For humans the value of 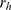 is taken from the thymic output of patients in their early 20s [19].

**Table 1:** Numerical values for memory T cells in mice and humans.

Parameter Values							
		Human			Mouse		
Parameter	Units	IL-15	IL-7	Refs	IL-15	IL-7	Refs
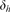	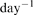	1/102	1/68	[19]	1/17	1/10	[20]
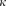	cells	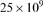	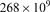	[23], [19]	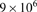	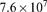	[19]
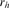	cells/day	0	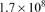	[16], [19]	0	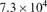	[19]

The values of 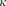 for IL-7 are the sum of naive and memory CD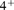 and naive CD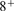 T cells. For humans the fraction of cell types in the blood was taken from Vrisekoop [19] and extrapolated to the total body using total cell numbers from Ganusov [23]. For mice, cell numbers in the spleen were taken from Vrisekoop [19] and extrapolated to whole body based on estimates of 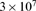 naive CD8 T cells in the whole body. The 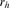 value for mice is taken from measurements of specific memory attrition, while for humans it is estimated from thymic output. Because we are interested in attrition rates the indirect measure provides better modeling accuracy.

The formula for the homeostatic equilibrium 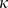 in the IL-7 niche is more complicated than for CD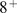 memory T cells due to the homeostatic influx 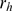. The total number of cells based on the parameters of the model is:
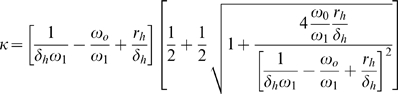
(22)

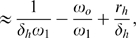
(23)where as with Eq. 18 the approximate form is valid if the expected life time is greater than the fastest possible time to division, 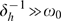. These two terms are observed to be approximately 10 days [20] and 12 hours, respectively [21].

We also have the form for the expected number of cells in the niche when the influx from infection 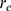 is non-zero:
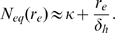
(24)This gives us the conditions for the suppression of autoreactive CD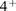 memory. From Eq. 1:
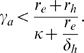
(25)


There are two additional effects which arise from the shared niche between the naive and Th1 and Th2 memory populations: the attrition of naive populations by inclusion of new memory, and a uniform suppressive effect, rather than a delicate balance of the Th1/Th2 ratio. The delicate balance for Th1/Th2 ratio is another proposed mechanism to explain the hygiene hypothesis.

The naive population typically contains many clones of small number. In a niche shared with memory cells, naive cells experience passive attrition. This results in the elimination of some naive sub-populations. This thinning of the naive repertoire has previously been studied [13], [22] and is a major contribution to immunosenescence. The separation of the memory and naive niches, as in the case of CD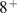 T cells, prevents this effect.

We have assumed here that the action of IL-7 stimulates division in the same way as IL-15. This assumption is based on the similarity and commonality between the receptor molecules for both interleukins. However, if IL-7 acts by inhibiting apoptosis, the boundary between suppression and proliferation (Eq. 19 and 25 and Fig. 3 and 6) will have the same low 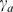 behavior but will have a vertical asymptote at the faster rate 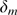, the rate of cell death in the absence of IL-7.

## Results

The conditions for suppression of autoreactive cells by passive attrition are plotted in Figure 6. Suppression depends on the antigenic stimulation rate 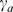 (horizontal axis) and the rate of new memory incorporation 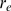 (vertical axis). The conditions are specified mathematically by Eq. 19 for CD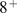 memory T cells and (under the assumption that IL-7 has an effect similar to IL-15) Eq. 25 for CD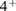 memory T cells. Figure 6 presents predictions for humans (solid lines) and mice (dashed lines) for both the CD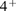 memory T cells competing for IL-7 (Red) and the CD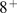 memory T cells competing for IL-15 (Black). The physiological parameter values for the terms in these equations are found in Table 1. We have also shown that this mechanism is not unique to these cell types but that it applies to any cell type under homeostatic regulation.

The characteristic features of Figure 6 are discussed in the Model section. Autoreactive cells with 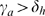 (right hand side of Figure 6) would receive antigenic stimulation at a rate rapid enough to maintain the population in the absence of homeostatic survival factors. Passive attrition would not be able to suppress these cells for this reason. Presumably, these cells are removed by negative selection as they are the most autoreactive, and if they were not removed autoimmune disease would be much more common.

If the initial growth rate of an autoimmune disease is measured, these charts will tell if the autoreactive population can be suppressed, and if so, what rate of new memory incorporation is required. Alternatively, if an environment is characterized by measuring passive attrition rates, this chart will show what autoreactivities will be suppressed by those environmental conditions.

The assumption that the action of IL-7 stimulates division in the same way as IL-15 is based on the similarity and commonality between the receptor molecules for both interleukins. If IL-7 acts by inhibiting apoptosis, the boundary between suppression and proliferation (Eq. 19 and 25 and Fig. 3 and 6) will have the same low 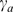 behavior but will have a vertical asymptote at the faster rate 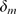, the rate of cell death in the absence of IL-7.

## Discussion

The results of Eq. 19, 25 and Fig. 6 can be used to limit the risk of autoimmune disease. Negative selection provides tolerance to autoimmune diseases by removing the most autoreactive lymphocytes before they mature. If all cells with autoreactivity above a threshold value 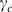 are removed by negative selection, this model would tell us the influx of new memory (i.e. infection rate) required to suppress all autoreactivities less than 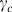, the autoreactive cells that may escape negative selection. In this manner the two tolerizing mechanisms in combination can cover the entire spectrum of 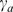 values.

**Figure 7:**
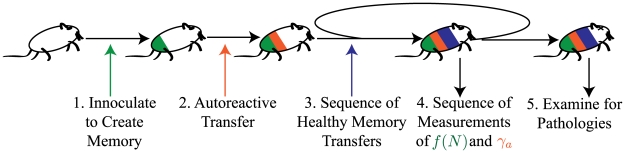
Schematic of proposed experimental protocol. Individual steps 1–5 are described in more detail in the corresponding enumerated list in the text.

This quantitative model makes experimentally testable predictions. We propose the following experiment illustrated in Figure 7. This will measure the threshold of cell influx needed to suppress autoimmune disease and can be compared with the predictions of Figure 6. Experimental Protocol:

1. Create an antigen specific memory population. This will be used for a direct measurement of the attrition rate 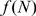.
2. Active transfer of a small population of autoreactive cells. This population should be large enough to induce autoimmune disease in an animal in a sterile environment.
3. Time-series of new memory inclusions. Active transfer of non-autoreactive memory cells, not specific for the antigen used in Step 1, or induction of new memory cells to create a range of 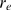 values.
4. Repeated measurements during time-series of new memory inclusions. Count how many of the initial specific memory population 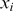 are present to measure the attrition rates. Count the autoreactive cell number 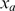. In the case of 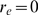 the measurement of autoreactive cell number will allow calculation of 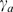 for that autoreactive clone. The best choice of measurement location (blood, spleen, lymph node or other tissue) is likely to be specific to the chosen autoimmune disease. Cell counts made where populations are the largest will yield higher accuracy in rate measurement.
5. Examine animals for pathology of transferred autoimmune disease.

Measuring the passive attrition rate by a time series count of the specific memory population created in step 1 will eliminate any uncertainties associated with the active transfer process. Inducing passive attrition through infection or immunization could also complicate the experiment through bystander activation or by activating other inflammatory and tolerizing aspects of the immune response. It would be best therefore, to first perform the experiment with active transfer of memory cells in Step 3 to eliminate the possibility of these complications.

Active transfer of memory cells eliminates other possible suppressive mechanisms. Performing the above experiment using infections will show the extent to which other mechanisms may suppresses autoimmune disease through pathogenic infection. Similar results for the active transfer and infection experiments would indicate that passive attrition is the dominant mechanism in nature for suppression of immune disorders by pathogen.

Measuring passive attrition rates presents a method of characterizing an environment. Though performing the above experiment for humans may be difficult, looking at regional trends in passive attrition rates and comparing them with prevalence of autoimmune disorders should yield an anti-correlation between the two quantities. The passive attrition rates in humans in different regions could most easily be measured by looking at the numbers of cells specific to smallpox vaccines, as this sub-population of cells is not likely to have been re-stimulated. The attrition rates of small pox immunity have been measured for vaccinia virus [24]. To our knowledge a regional study has not been performed.

It has also been suggested that the beneficial effects of exposure to infectious diseases are most important for children [1]. There are two effects that may contribute to this: the expansion of the niche may favor autoreactive growth, and the higher flux of new cells from the thymus increases the rate of new autoreactive cells entering the niche. Mathematical modeling of the effects of passive attrition on autoreactive populations under these conditions is the subject of current work.

The competition for survival factors (i.e. passive attrition) is one of several proposed mechanisms by which infectious diseases may confer protection from immune system disorders. Other proposed mechanisms include Th1/Th2 balance, and generation or activation of regulatory T cells by infection.

Th1 and Th2 cells each have a suppressive effect on the other. A system out of balance in population numbers might result in one cell type being unregulated by the other. Th1 cells are implicated in autoimmune diseases while Th2 cells are a component of allergic disease. If Th1/Th2 balance is the mechanism generating a suppression of immune system disorders, we expect allergy and autoimmune prevalence to be inversely correlated. However, this is not the case [1]. It has also been observed that individuals with diabetes or rheumatoid arthritis have a higher incidence of atopic disease [1], [25], [26], in contradiction to the hypothesized Th1/Th2 balance dynamic. This observation is in agreement with the predictions of passive attrition. Suppression by passive attrition is independent of the method of new cell introduction, whether it is from a Th1 or a Th2 response. It only depends on the number of new cells created that are competing for IL-7.

Bystander suppression by regulatory T cells in response to infections is a compelling hypothesis. These cells have many possible mechanisms of suppression, and characterization of these cell types is still in its infancy. It has been shown that the suppressive effects conferred by killed bacteria persist in IL-10 and IL-4 knockout NOD mice [1], [27]. These suppressive cytokines however are only two of the several possible mechanisms that regulatory T cells may be using to suppress immune system disorders so this mechanism can not currently be ruled out.

Mathematical models of the expected level of protection conferred by each of these mechanisms will give rise to insight testable predictions that will reveal which mechanisms are dominant. Passive attrition should be capable of suppressing small populations of autoreactive cells, but it comes with the price of accelerated loss of immunity. This tradeoff is one of many the immune system must balance.
